# Short‐course daily isoniazid and rifapentine for latent tuberculosis infection in people living with HIV who received coformulated bictegravir/emtricitabine/tenofovir alafenamide

**DOI:** 10.1002/jia2.25844

**Published:** 2021-11-25

**Authors:** Bo‐Huang Liou, Chih‐Ning Cheng, Ya‐Ting Lin, Yu‐Jou Lin, Yu‐Chung Chuang, Kuan‐Yin Lin, Wen‐Chun Liu, Shu‐Wen Lin, Ching‐Hua Kuo, Hsin‐Yun Sun, Chien‐Ching Hung

**Affiliations:** ^1^ Department of Internal Medicine Hsinchu MacKay Memorial Hospital Hsinchu City Taiwan; ^2^ School of Pharmacy National Taiwan University Taipei Taiwan; ^3^ Department of Pharmacy National Taiwan University Hospital and National Taiwan University College of Medicine Taipei Taiwan; ^4^ Department of Internal Medicine National Taiwan University Hospital and National Taiwan University College of Medicine Taipei Taiwan; ^5^ Graduate Institute of Clinical Pharmacy National Taiwan University College of Medicine Taipei Taiwan; ^6^ Department of Tropical Medicine and Parasitology National Taiwan University College of Medicine Taipei Taiwan

**Keywords:** rifamycin, integrase strand transfer inhibitor, drug–drug interaction, trough concentration, protein‐adjusted 95% effective concentration

## Abstract

**Introduction:**

Short‐course preventive therapy with 1‐month course of daily administration of isoniazid (300‐mg) plus rifapentine (600‐mg) (1HP) and 3‐month course of weekly administration of isoniazid (900‐mg) plus rifapentine (900‐mg) (3HP) has higher completion rates than 9‐month course of daily isoniazid (9H) for individuals with latent tuberculosis infection (LTBI). We aimed to evaluate the effect, safety and tolerability of 1HP in people living with HIV (PLWH) and LTBI who received coformulated bictegravir/emtricitabine/tenofovir alafenamide (BIC/FTC/TAF).

**Methods:**

PLWH testing positive by interferon‐gamma release assay and having received BIC/FTC/TAF for >2 weeks with plasma HIV RNA load (PVL) <200 copies/ml were enrolled. BIC trough plasma concentrations and cytokine profiles were determined before the first dose (day 1/baseline), 24 h after the 14th (day 15) and 28th (day 29) doses of 1HP. PVL were determined on days 15 and 29 of 1HP and every 3 months subsequently after discontinuation of 1HP.

**Results:**

From November 2019 to December 2020, 48 PLWH with LTBI were enrolled. One participant (2.1%) discontinued 1HP on day 15 due to fever and generalized rashes with PVL of 72 copies/ml, which was <50 copies/ml in three subsequent determinations while on BIC/FTC/TAF over the 12 months of follow‐up. The percentages of BIC trough plasma concentrations above the protein‐adjusted 95% effective concentration (paEC_95_ = 162 ng/ml) were 56.3% and 37.0% on days 15 and 29, respectively. The percentage of PVL <200 copies/ml was 91.7% on day 15, 97.8% on day 29 and 100% at both months 3 and 6. After a median observation of 52 weeks (interquartile range, 51–55), all participants continued BIC/FTC/TAF with a median PVL of 20 copies/ml (range 20–331). Except for the participant who discontinued 1HP because of allergic reactions, none of the participants had relevant symptoms or increases of the cytokine levels assessed between baseline and days 15 and 29 of 1HP.

**Conclusions:**

BIC/FTC/TAF in combination with 1HP was well tolerated with a high completion rate. BIC trough plasma concentrations were significantly decreased with concurrent use of 1HP among PLWH with LTBI. While transient viral blips were observed during 1HP without causing subsequent treatment failure, such combination should be applied with caution.

## 1 INTRODUCTION

Tuberculosis (TB) is the leading cause of morbidity and mortality among people living with HIV (PLWH) globally. In 2019, 820,000 TB cases occurred with 208,000 deaths among PLWH [1]. HIV infection increases the risk of developing TB among individuals with latent TB infection (LTBI) by more than 20 times compared with general population [1, 2]. Previous studies have demonstrated that TB preventive therapy reduced TB incidence and mortality among PLWH in area of moderate to high endemicity, irrespective of the status of tuberculin skin test or results of interferon‐gamma release assay (IGRA) [3], and independently of antiretroviral therapy (ART) [4, 5, 6, 7]. The World Health Organization (WHO) guidance recommends TB preventive therapy for PLWH regardless of CD4 lymphocyte counts or plasma HIV RNA loads (PVLs) [2].

Recent data from BRIEF‐TB trial demonstrated that 1‐month regimen of daily isoniazid plus rifapentine (1HP) was comparable to 9‐month regimen of daily isoniazid (9H) in terms of prevention against TB and achieved a higher treatment completion rate and lower rate of drug discontinuation related to hepatotoxicity in PLWH [8]. This rifapentine‐based, ultra‐short course of TB preventive therapy could be an important strategy to control HIV‐related TB. TB preventive therapy and ART can act synergistically to reduce the risk of developing TB, and combination of both interventions is recommended for PLWH with LTBI [9]. However, potential drug–drug interactions between rifapentine and multiple antiretrovirals remain a concern [2].

Based on the pharmacokinetic studies derived from the BRIEF‐TB trial, daily rifapentine can be administered with efavirenz without clinically relevant reductions in the efavirenz plasma concentration or virologic suppression, but not with nevirapine because of a significant decrease of nevirapine trough concentrations [10, 11]. Recent studies suggested that both raltegravir and dolutegravir (DTG) can be used at a standard dose with rifapentine once weekly as a part of 3‐month course of isoniazid plus rifapentine regimen (3HP) [12, 13]. Rifapentine taken once weekly significantly increased DTG clearance by 36%, resulting in a 26% decrease in DTG area‐under‐the‐curve (AUC) concentration, but the geometric mean trough concentration was well above the protein‐adjusted 90% maximal inhibitory concentration (paIC_90_), and viral suppression could be maintained [12]. Nevertheless, the study of DTG in combination with 3HP in healthy subjects was terminated early because of the development of flu‐like syndrome and elevated aminotransferase and inflammatory cytokine levels in two of four participants after the third weekly dose [14]; however, this observation was not confirmed by another study of 60 PLWH concurrently received DTG‐based ART and 3HP [12].

Coformulated bictegravir/emtricitabine/tenofovir alafenamide (BIC/FTC/TAF) is recommended as one of the preferred regimens for most PLWH initiating ART and can also be used as a switch regimen [15, 16, 17]. The metabolism of BIC is primarily driven through hepatic cytochrome P450 isoenzyme 3A (CYP3A) oxidation and UDP‐glucuronosyltransferase 1A1 (UGT1A1) glucuronidation. In addition, BIC and TAF are both substrates of drug transport proteins, such as P‐glycoprotein (P‐gp) and breast cancer resistance protein (BCRP) [17, 18, 19]. Rifapentine, a rifamycin derivative, is known to be an inducer of CYP3A, UGT1A1, P‐gp, and BRCP, which may potentially increase the clearance of BIC and TAF. Co‐administration of BIC/FTC/TAF with rifapentine is not recommended by the manufacturer [18]. Whether rifapentine reduces BIC/FTC/TAF concentration among PLWH in a clinically relevant way remains unknown and is of great interest given the high potential value of 1HP for TB prevention and the important role of BIC/FTC/TAF in the modern era of HIV treatment.

This study aimed to investigate the sparse pharmacokinetics, safety, cytokine changes, tolerability and HIV suppression among PLWH who had LTBI and concurrently received BIC/FTC/TAF and 1HP in Taiwan, where the TB incidence was 37 per 100,000 population in 2019.

## 2 METHODS

### 2.1 Study setting and participants

PLWH in Taiwan are provided with free‐of‐charge combination ART and monitoring of PVL, CD4 lymphocyte counts, renal and liver functions, lipid profiles, and serologic markers of viral hepatitis and viral loads of hepatitis viruses according to the national HIV treatment guidelines [20]. In 2019, Taiwan Centers for Disease Control implemented a program of diagnosis and treatment of LTBI among PLWH by providing free‐of‐charge IGRA and treatment with 1HP, 3HP or 9H. The program required that LTBI treatment be conducted by HIV‐treating infectious disease specialists and case managers with a video directly observed approach with the use of smartphones.

The present study enrolled PLWH who tested positive for LTBI by IGRA and had received BIC/FTC/TAF for at least 2 weeks with PVL <200 copies/ml within 6 months of initiation of 1HP. PLWH with active TB or suspicion of active TB, a history of having received treatment for LTBI or TB, a known history of intolerance to antiretroviral regimens containing an integrase inhibitor or to isoniazid or rifapentine, genotypic resistance to integrase inhibitors or TFV, concurrent use of medications known to pharmacokinetically interact with BIC and pregnancy were excluded.

### 2.2 Treatment of LTBI

For participants receiving 1HP, rifapentine (Sanofi‐Aventis U.S. LLC., Bridgewater, NJ, USA) was administered at a daily dose of 300 mg for PLWH with a weight of <35 kg, 450 mg for those with a weight of 35–45 kg and 600 mg for those with a weight of >45 kg; plus isoniazid (Cadila Pharmaceuticals Limited, India) at a daily dose of 300 mg [8]. The 1HP treatment course was 28 days.

### 2.3 Study end points

The primary end points were the proportion of study participants maintaining BIC trough plasma concentrations above the protein‐adjusted 95% effective concentration (paEC_95_) of 162 ng/ml [18, 21] on days 15 and 29, and that of participants maintaining PVL <200 copies/ml at the end of 1HP. The secondary end points were the safety of 1HP, discontinuation rate of 1HP and virologic rebound (>1000 copies/ml) during the 6‐month follow‐up duration. The inhibitory quotient (IQ), the ratio of drug exposure to viral susceptibility [22], was defined as the ratio of the BIC trough concentration to paEC_95_ of 162 ng/ml. The study was approved the Research Ethics Committee of the hospital (NTUH‐REC No. 201911034RINB), and the participants gave written informed consent before participation in the study.

### 2.4 Determinations of trough plasma concentrations of BIC, FTC, TFV, 1HP and cytokines

BIC, FTC, TFV and 1HP trough plasma concentrations were determined with the use of ultra‐high performance liquid chromatography‐tandem mass spectrometry (UHPLC‐MS/MS) system before the first dose (day 1/baseline), 24 h after the 14th (day 15) and 28th (day 29) doses of 1HP, while isoniazid peak concentrations were determined 3 h after the 1st and 15th doses of 1HP. PVL was determined within 6 months before and 2 weeks after 1HP initiation (day 15), end of 1HP (day 29), and 3 and 6 months subsequently after discontinuation of 1HP. Cytokine levels (interferon [IFN]‐gamma, interleukin [IL]‐10, IL‐12p40, IL‐12p70, IL‐15, IL‐17A, IL‐2, IL‐5, IL‐6, IL‐8, interferon gamma‐induced protein [IP]‐10, monocyte chemoattractant protein [MCP]‐1, macrophage inflammatory protein [MIP]1B and tumour necrosis factor [TNF]‐alpha) were determined with the use of MILLIPLEX® _MAP_ Kit on days 1 (before the 1st dose of 1HP), 15 and 29 (24 h after the 14th and 28th doses of 1HP).

The preparation procedure of the blood samples was as follows. One hundred μl of plasma samples were spiked 40 μl of internal standards (1.1 ppm of bictegravir‐^15^N, d2, isoniazid‐d4 and rifapentine‐d8), followed by adding 300 μl of acetonitrile for protein precipitation. The mixture was vortexed by Geno/Grinder 2010 (SPEX, Metuchen, NJ, USA) at 1000 rpm for 2 min. The sample was centrifuged at 15,000 *g* for 15 min at 4 °C. The supernatant was collected and filtered through a 0.22‐μm regenerated cellulose membrane. A 10‐μl filtered solution was added with 150 μl of 50% acetonitrile for dilution, followed by UHPLC‐MS/MS analysis. The UHPLC‐MS/MS method is provided in Section S1. The validation data showed that the coefficient of determination was greater than 0.998 for three calibration curves. The accuracy was within 100±15%, and precision was within relative standard deviation of 15%. Quality control samples were utilized to monitor the system performance for every 10 real sample injections during the analytical procedure. The relative standard deviation of three target drug signals in all of the final quality control samples was within 5%.

For the analysis of FTC and TFV, 50 μl of plasma samples were spiked 50 μl of internal standards (tenofovir‐d5 and emtricitabine‐^13^C^15^N_2_), followed by adding 700 μl of 50% methanol for protein precipitation. The sample was centrifuged at 18,000 *g* for 5 min at 4 °C. All the supernatant was dried under the gentle stream of nitrogen. The residue was reconstituted with 200 μl of 50% methanol and filtered through a 0.22‐μm regenerated cellulose membrane. The sample was directly introduced into the UHPLC–MS/MS system. The UHPLC‐MS/MS method is provided in Section S2.

### 2.5 Statistical analysis

All analyses were performed using Statistical Program for Social Sciences (SPSS Statistics Version 21, IBM Corp., Armonk, NY, USA). Categorical variables were compared using *X* ^2^ or Fisher's exact test and non‐categorical variables using Student's *t* test or Mann–Whitney *U* test. Given the small case number of the participants, non‐parametric tests were used.

## 3 RESULTS

**Figure 1:**
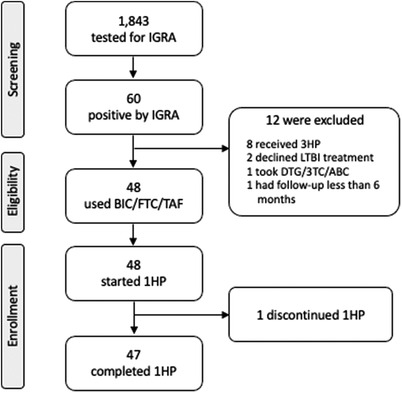
Study flow. Abbreviations: 1HP, 1‐month course of daily isoniazid plus rifapentine; 3HP, 3‐month course of weekly isoniazid plus rifapentine; BIC/FTC/TAF, bictegravir/emtricitabine/tenofovir alafenamide; DTG/3TC/ABC, dolutegravir/lamivudine/abacavir; IGRA, interferon‐gamma release assay; LTBI, latent tuberculosis infection.

From November 2019 to December 2020, a total of 1843 PLWH underwent IGRA testing and 60 (3.3%) tested positive; 48 PLWH with LTBI agreed to participate in the study and received 1HP as their LTBI treatment and BIC/FTC/TAF as their ART, while 8 received 3HP, 2 declined LTBI treatment, 1 took DTG/lamivudine/abacavir and 1 who received 1HP and BIC/FTC/TAF had follow‐up less than 6 months (Figure 1).

The demographic and clinical characteristics of the 48 participants are shown in Table 1. Their median age was 41.0 years, 97.9% were men and 89.6% were men who have sex with men. One participant (2.1%) developed fever and generalized rashes on day 15 and discontinued 1HP. His IFN‐gamma, IL‐6, TNF‐alpha and IL‐10 levels increased by five‐fold or more than those at baseline. His PVL was 72 copies/ml on day 15 and subsequently became <50 copies/ml at follow‐up on days 70, 163 and 357 after 1HP discontinuation while he continued BIC/FTC/TAF. The data were missing in two patients on day 29, four at month 3 and two at month 6 (Table 1). Fold‐changes of cytokine levels from the baseline on days 15 and 29 in 48 PLWH are shown in Table S1, and none had relevant symptoms concurrently.

**Table 1:** Demographic and clinical characteristics of 48 PLWH with latent tuberculosis infection who received daily isoniazid plus rifapentine (1HP) and BIC/FTC/TAF concurrently

Variables	Data
Age, median (IQR), years	41.0 (37.0–51.8)
Male sex, *n* (%)	47 (97.9)
Risk group for HIV transmission, *n* (%)	
Men who have sex with men	43 (89.6)
Heterosexuals	5 (10.4)
Before 1HP initiation	
Duration of ART, median (IQR), years	6.0 (4.0–10.8)
PVL <200 copies/ml, *n* (%)	48 (100.0)
PVL <50 copies/ml	47 (97.9)
CD4 >200 cells/mm^3^	46/47 (95.8)
AST, median (IQR), U/L	20.0 (18.0–24.0)
ALT, median (IQR), U/L	19.5 (13.3–26.8)
Total bilirubin, median (IQR), mg/dl	0.6 (0.5–0.7)
Day 15 of 1HP	
PVL <200 copies/ml, n/N (%)	44/48 (91.7)
PVL <50 copies/ml	35/48 (72.9)
AST, median (IQR), U/L	21.0 (18.0–27.0)
ALT, median (IQR), U/L	17.5 (12.3–25.0)
Total bilirubin, median (IQR), mg/dl	0.8 (0.7–1.0)
Day 29 of 1HP	
PVL <200 copies/ml, n/N (%)	46/46^a^ (100.0)
PVL <50 copies/ml	43/46^a^ (93.5)
AST, median (IQR), U/L	21.0 (18.0–26.0)
ALT, median (IQR), U/L	19.0 (13.0–27.0)
Total bilirubin, median (IQR), mg/dl	0.8 (0.7–0.9)
Month 3 after 1HP discontinuation	
PVL <200 copies/ml, n/N (%)	44/44^b^ (100.0)
PVL <50 copies/ml	43/44^b^ (97.7)
Month 6 after 1HP discontinuation	
PVL <200 copies/ml, n/N (%)	46/46^c^ (100.0)
PVL <50 copies/ml	46/46^c^ (100.0)

Abbreviations: 1HP, 1‐month course of isoniazid and rifapentine; ALT, alanine aminotransferase; ART, antiretroviral therapy; AST, aspartate aminotransferase; BIC, bictegravir; FTC, emtricitabine; IQR, interquartile range; PLWH, people living with HIV; PVL, plasma HIV RNA load; TAF, tenofovir alafenamide.

^a^ On day 29, two participants, including the one who discontinued daily isoniazid plus rifapentine on day 15 due to allergic reactions, missed PVL testing.

^b^ At month 3, four participants missed PVL testing.

^c^ At month 6, two participants missed PVL testing.

Before initiation of 1HP, the median duration of combination ART was 6.0 years, and 25 (52.1%) PLWH had undergone HIV genotypic resistance testing with 21 (84.0%) before and 4 (16.0%) after ART initiation. No major resistance mutations to integrase inhibitors and nucleoside/nucleotide reverse transcriptase inhibitors were identified. The percentages of PLWH with PVL <200 and <50 copies/ml were 100.0% and 97.9%, respectively, before 1HP was initiated. Their liver‐function tests were within normal limits before initiation of 1HP and did not change significantly on both days 15 and 29. However, the percentages of PVL <200 and <50 copies/ml declined to 91.7% and 72.9%, respectively, on day 15 of 1HP, which subsequently changed to 100% and 93.5%, respectively, on day 29. The percentages of PVL <200 and <50 copies/ml returned to 100.0% and 97.7%, respectively, at month 3, and both 100.0% at month 6 (Table 1). After a median observation of 52 weeks (interquartile range [IQR], 51–55), all 48 participants continued to receive BIC/FTC/TAF with a median plasma HIV RNA of 20 copies/ml (range, 20–331). The clinical characteristics of the participants who failed to achieve PVL <50 copies/ml on days 15 and 29 are shown in Table S2.

Sequential changes of BIC, FTC and TFV trough plasma concentrations at baseline, and on days 15 and 29 are shown in Table 2. With the co‐administration of 1HP, BIC trough concentrations decreased on days 15 and 29; the median BIC trough concentrations were 3927.31 ng/ml at baseline, 182.32 on day 15 and 138.54 on day 29 (Table 2). BIC trough concentrations decreased substantially from baseline to day 15 by 94.6% (range, 55.3–99.5%) and to day 29 by 95.5% (range, 57.2–99.2%). No significant changes were observed in sequential plasma FTC and TFV trough concentrations at baseline and on days 15 and 29 (Table 2).

**Table 2:** Sequential trough plasma concentrations of bictegravir (BIC), emtricitabine (FTC) and tenofovir (TFV) at baseline, days 15 and 29

	Baseline	Day 15	Day 29
Number of patients with available data	46^a^	48	46^b^
BIC trough concentrations, median (IQR), ng/ml	3927.31 (2255.25–5254.57)	182.32 (125.97–256.38)	138.54 (108.26–207.88)
BIC geometric mean concentration, ng/ml	3356.45	181.48	159.07
BIC geometric mean ratio^c^	NC	0.054	0.047
FTC trough concentration, median (IQR), ng/ml	70.24 (54.70–95.87)	64.23 (48.40–84.75)	60.78 (50.96–78.40)
FTC geometric mean concentration, ng/ml	75.81	65.16	65.11
FTC geometric mean ratio^c^	NC	0.86	0.86
TFV trough concentration, median (IQR), ng/ml	12.68 (10.19–15.78)	11.34 (9.38–13.90)	11.84 (8.82–13.77)
TFV geometric mean concentration, ng/ml	12.80	11.36	11.31
TFV geometric mean ratio^c^	NC	0.89	0.88

^a^ At baseline, two participants missed the testing of BIC/FTC/TFV trough plasma concentrations.

^b^ Including the participant who discontinued daily isoniazid plus rifapentine on day 15 due to allergic reactions and another participant who missed the testing of BIC/FTC/TFV trough plasma concentrations.

^c^ Geometric mean ratio, the ratio of geometric mean concentration on day 15 or day 29 to that at baseline.

Abbreviations: IQR, interquartile range; NC, not calculable.

The percentage of BIC trough concentrations above 162 ng/ml (BIC paEC_95_) was 100% before 1HP initiation, which dropped to 56.2% (27/48) and 37.0% (17/46) on days 15 and 29, respectively (Table 3). Of the 21 (43.8%) participants with BIC trough concentrations below 162 ng/ml on day 15, the percentages of PVL <200 and <50 copies/ml were 95.2% and 81.0%, respectively, and the median PVL was 20 copies/ml (range, 20–216). Of the 29 (63.0%) PLWH with BIC trough concentrations below 162 ng/ml on day 29, the percentages of PVL <200 and <50 copies/ml were 96.6% and 89.7%, respectively, and the median PVL was 20 copies/ml (range, 20–205).

**Table 3:** The relationship between plasma HIV RNA load and bictegravir (BIC) trough concentration above or below 162 ng/ml in 48 participants with BIC trough concentrations determined

	All PLWH	Participants with BIC trough concentrations>162 ng/ml	Participants with BIC trough concentrations<162 ng/ml	*p* value
Baseline, *n*/*N* (%)		46/46^a^ (100)	0/46^a^ (0)	
PVL, median (range), copies/ml	20 (20–85)	20.0 (20–85)	NA	NC
PVL <200, *n*/*N* (%)	46/46 (100)	46/46 (100)	NA	NC
PVL <50	45/46 (97.8)	45/46 (97.8)	NA	NC
Day 15, *n*/*N* (%)		27/48 (56.2)	21/48 (43.8)	
PVL, median (range), copies/ml	20 (20–423)	20.0 (20–423)	20 (20–216)	0.649
PVL <200, *n*/*N* (%)	44/48 (91.7)	24/27 (88.9)	20/21 (95.2)	0.621
PVL <50	35/48 (72.9)	18/27 (66.7)	17/21 (81.0)	0.338
Day 29, *n*/*N* (%)		17/46^b^ (37.0)	29/46^b^ (63.0)	
PVL, median (range), copies/ml	20 (20–205)	20 (20–97)	20 (20–205)	0.259
PVL <200, *n*/*N* (%)	45/46 (97.8)	17/17 (100)	28/29 (96.6)	0.999
PVL <50	42/46 (91.3)	16/17 (94.1)	26/29 (89.7)	0.999

^a^ At baseline, two participants missed the testing for BIC trough concentrations.

^b^ On day 29, two participants had missing data, including the participant who discontinued daily isoniazid plus rifapentine on day 15 due to allergic reactions and another participant who missed the testing for BIC trough concentration.

Abbreviations: NA, not applicable; NC, not calculable; PVL, plasma HIV RNA load.

Comparisons of clinical characteristics and BIC, FTC and TFV trough plasma concentrations between PLWH with and without PVL <50 and <200 copies/ml on day 15 are shown in Table 4. There were no specific factors found to be associated with a PVL <50 and <200 copies/ml on day 15. The concentrations of isoniazid and rifapentine are shown in Table S3. There were no significant changes of isoniazid and rifapentine concentrations that were determined on days 1, 15 and 29.

**Table 4:** Comparisons of clinical characteristics and bictegravir (BIC), emtricitabine (FTC) and tenofovir (TFV) trough plasma concentrations (A) between participants with plasma HIV RNA load <50 copies/ml and those with plasma HIV RNA load ≥50 copies/ml on day 15 and (B) between participants with plasma HIV RNA load <200 copies/ml and those with plasma HIV RNA load ≥200 copies/ml on day 15

(A)			
Variables	Participant with PVL <50 copies/ml	Participant with PVL ≥50 copies/ml	*p* value
Participant No.	35	13	
Baseline			
Duration of ART, median (IQR), years	8.00 (5–12)	6.00 (5.00–9.00)	0.185
BIC concentration, median (IQR), ng/ml	3873.08 (2169.04–5279.63)	4150.32 (2237.72–5075.56)	0.810
BIC IQ, median (IQR)	23.91 (13.39–32.59)	26.62 (13.81–31.33)	0.810
FTC concentration, median (IQR), ng/ml	71.38 (54.78–94.19)	70.24 (55.29–96.58)	0.887
TFV concentration, median (IQR), ng/ml	12.94 (10.14–15.55)	12.17 (10.30–15.92)	0.851
Day 15			
BIC concentration, median (IQR), ng/ml	165.02 (111.7–235.01)	217.02 (135.18–375.70)	0.188
Percentage of BIC concentration >162 ng/ml, *n* (%)	18 (51.4)	9 (69.2)	0.338
BIC IQ, median (IQR)	1.02 (0.69–1.45)	1.34 (0.83–2.32)	0.188
FTC concentration, median (IQR), ng/ml	64.53 (49.41–85.21)	66.97 (53.44–87.70)	0.917
TFV concentration, median (IQR), ng/ml	11.53 (9.67–13.98)	11.71 (9.54–14.07)	0.917


(B)			
Variables	<200 copies/ml	≥200 copies/ml	*p* value
Patient No.	44	4	
Baseline			
Duration of cART, median (IQR), years	8.00 (5.00–12.00)	5.50 (2.00–7.50)	0.152
BIC concentrations, median (IQR), ng/ml	3870.57 (2255.25–5254.57)	4595.49 (2634.97–5234.73)	0.612
BIC IQ, median (IQR)	23.89 (13.92–32.44)	28.37 (16.27–32.31)	0.612
FTC concentration, median (IQR), ng/ml	70.24 (54.70–95.87)	80.40 (62.50–96.94)	0.170
TFV concentration, median (IQR), ng/ml	12.68 (10.19–15.78)	13.54 (11.83–15.94)	0.269
Day 15			
BIC concentration, median (IQR), ng/ml	175.59 (114.71–256.38)	224.61 (146.72–344.84)	0.482
Percentage of BIC concentration >162 ng/ml, *n* (%)	24 (54.5)	3 (75.0)	0.621
BIC IQ, median (IQR)	1.08 (0.71–1.58)	1.39 (0.91–2.13)	0.482
FTC concentration, median (IQR), ng/ml	64.23 (48.40–84.75)	73.72 (57.59–88.82)	0.117
TFV concentration, median (IQR), ng/ml	11.34 (9.38–13.90)	12.75 (10.18–14.78)	0.737

*Note*: IQ defined as the ratio of the BIC trough concentration to the protein‐adjusted 95% effective concentration of 162 ng/ml.

Abbreviations: ART, antiretroviral therapy; IQR, interquartile range; IQ, inhibitory quotient.

The completion rate of 1HP treatment was 97.9% and the spectrum of solicited adverse events (AEs) are presented in Tables S4 and S5. Drug‐related AEs occurred in 81.3% of 48 PLWH receiving 1HP with predominantly flu‐like symptoms (68.8%) followed by cutaneous reactions (45.8%). The AEs were mainly of grade 1 (37.5%) and 2 (29.2%) in severity, and there were no grade 4 AEs. The median interval from 1HP initiation to the onset of AEs was 2 days (IQR, 1–11).

## 4 DISCUSSION

While high‐quality evidence and recommendations from the WHO support the use of TB preventive therapy among PLWH, there is a knowledge gap regarding the optimal antiretroviral options because of the concerns about drug–drug interactions between rifapentine and contemporary antiretroviral agents, especially integrase inhibitor‐based (BIC and DTG) regimens. In this study consisting of virologically suppressed PLWH with LTBI, we found that the 28‐day course of 1HP was safe and well tolerated for participants receiving BIC/FTC/TAF. The BIC trough concentrations, however, were significantly reduced by the concurrent administration of rifapentine. The proportion of participants with BIC trough plasma concentrations above 162 ng/ml was <60% on days 15 and 29 over the treatment course of 1HP, which resulted in an increased rate of on‐treatment virologic blips. The proportions of patients with PVL ≥50 copies/ml were 27.1% on day 15 and 6.5% on day 29. Due to the small case number of participants enrolled in this study, no specific factors associated with PVL ≥50 copies/ml on day 15 were identified. Nevertheless, after completion of 1HP, nearly all participants re‐achieved virologic suppression with BIC/FTC/TAF at months 3 and 6 and subsequent follow‐up for a median of 52 weeks (Table 1 and Table S2).

Viral blips, defined as PVL ≥50 copies/ml preceded and followed by PVL <50 copies/ml after the confirmed suppression (two consecutive PVL values of <50 copies/ml), developed in an average of 1.3% of participants treated with BIC/FTC/TAF, DTG/lamivudine/abacavir (DTG/3TC/ABC) or DTG plus FTC/TAF who had a viral blip per study visit in the clinical trials [23]. However, virologic suppression at week 144 of the BIC/FTC/TAF group was not affected by blips, and no emergence of resistance‐associated mutations was detected [23]. In our study, none of the participants had to discontinue BIC/FTC/TAF subsequently because of virologic failure, despite viral blips detected when 1HP was concurrently taken with BIC/FTC/TAF.

Unlike DTG trough concentrations achieved that were higher than paIC_90_ with co‐administration with 3HP, concurrent administration of once‐daily rifapentine and BIC/FTC/TAF resulted in only 56.2% and 37.0% of the study participants attaining paEC_95_ of BIC on days 15 and 29, respectively, after 1HP initiation, which might have contributed to the temporary loss of therapeutic effect observed. The finding that the measured BIC trough concentrations with concurrent 1HP remained highly in excess of the half‐maximal effective concentration (EC50) of 1.1 ng/ml for wild‐type HIV‐1 and the fact that BIC exhibits a high genetic barrier to emergence of resistance‐associated mutations explain that the short‐term viral blips observed in this study did not lead to overt virologic failure (>1000 copies/ml) [19]. In PLWH taking DTG‐based ART, 3HP can be used, but the pharmacokinetic impact of 1HP on DTG is yet unknown. To date, only efavirenz was documented to be co‐administered with 1HP without clinically meaningful reductions in efavirenz concentrations or adverse impact on virologic suppression [10].

The impact of daily rifapentine on the plasma concentrations of BIC, FTC, TFV and tenofovir diphosphate (TFV‐DP) and virologic responses had not been evaluated among PLWH before. In the study enrolling 30 HIV‐negative participants by Arora and colleagues, combination of BIC/FTC/TAF with weekly rifapentine is not recommended because the BIC trough concentrations were decreased by 35–85% following daily administration of BIC/FTC/TAF and weekly rifapentine compared with those achieved by daily BIC/FTC/TAF alone; however, there are no clinically significant changes in the plasma concentrations of FTC, TAF, TFV and TFV‐DP [24]. Rifampin is a more potent inducer of CYP3A than rifapentine. Following co‐administration of rifampin and TAF, intracellular TFV‐DP concentrations were reduced significantly, but remained 4.21‐fold higher than those achieved by tenofovir disoproxil fumarate [25].

The extent of reduction of BIC trough concentration (94.6–95.5%) observed in our study of PLWH was greater than that in healthy volunteers in the study by Arora and colleagues [24], which might be related to higher overall weekly rifapentine exposure in our study (2100–4200 mg for weekly rifapentine dose of 1HP vs. 750–900 mg for weekly rifapentine of 3HP). However, nearly all participants achieved virologic suppression with BIC/FTC/TAF at months 3 and 6 and subsequent follow‐up. Furthermore, the percentages of subjects with PVL <50 copies/ml remained similar (97.4–99.1%) across the exposure quartiles in two phase 3 registrational studies [26]. Given that no BIC exposure–efficacy relationship exists [26], clinicians and patients should carefully weigh the benefits of co‐administration of short‐course rifapentine and BIC/FTC/TAF against the potential risks of viral replication.

Our completion rate of 1HP (97.9%) was consistent with that of previous study [8]. The high completion rate could be partially attributed to the implementation of video directly observed treatment program of LTBI with the use of smartphones in Taiwan. It could also be attributed to the high safety profile of combination of BIC/FTC/TAF and 1HP regimen. Though flu‐like symptoms and cutaneous reactions were reported by 68.8% and 45.8% of the participants, respectively, most adverse events (AEs) were of low grades and self‐limited. Only one participant discontinued 1HP on day 15 due to fever and generalized rashes with elevations of proinflammatory cytokines, and no other participants had cytokine changes with relevant clinical symptoms concurrently. The mechanism of cytokine‐mediated systemic adverse reactions observed in the DTG and 3HP study of four healthy subjects was unclear [14]. It could be related to robust immune reactions seen in HIV‐negative healthy volunteers, which may be less likely to occur in PLWH [12], or probably a chance finding.

Our study has several limitations. First, the participants had good adherence to ART given the virologically suppressed status before directly observed co‐administration of LTBI treatment and 1HP. Our findings may not be generalizable to all PLWH, whose adherence may be reduced by concurrent administration of 1HP that was self‐administered without supervision. Moreover, the findings of safety profile and virologic response cannot be generalized to antiretroviral‐naïve PLWH. Second, we only determined the trough concentrations at baseline, and on days 15 and 29; our sparse pharmacokinetic study did not assess the other pharmacokinetic parameters, such as maximal concentration, AUC concentration and oral clearance. Further studies recruiting a larger number of subjects with intensive pharmacokinetic assessment are needed to determine the steady‐state pharmacokinetics and other pharmacokinetic parameters. Rifapentine concentrations were found to be lower in PLWH than those in HIV‐negative population at the same dose [27], and the results of investigating BIC and 1HP interaction in healthy volunteers should be interpreted with caution. Third, our study was a single‐arm study that was designed to examine whether 1HP could have any impact on PLWH with LTBI who concurrently received BIC/FTC/TAF. The impact of 3HP on the pharmacokinetics and tolerability of virologic response to BIC/FTC/TAF among PLWH remains to be examined. Lastly, we assessed only trough plasma concentrations of TFV and FTC, not intracellular TFV‐DP concentrations. Nevertheless, our mean TFV trough plasma concentration of 1HP and BIC/FTC/TAF on day 15 was similar to that of TAF alone on day 10 [28].

## 5 CONCLUSIONS

In conclusion, 1HP combined with coformulated BIC/FTC/TAF was safe and well tolerated in PLWH with LTBI. While BIC trough plasma concentrations were significantly reduced by co‐administered rifapentine, which led to viral blips during the 1‐month period of co‐administration, no subsequent treatment failure developed. Such combination should be applied with caution.

## COMPETING INTERESTS

C‐CH has received research support from Janssen, Merck, Gilead Sciences and ViiV and speaker honoraria from Abbvie, Bristol‐Myers Squibb, Gilead Sciences and ViiV, and served on advisory boards for Gilead Sciences, Janssen, ViiV and Abbvie. H‐YS has received research support from Gilead. Other authors have no competing interest to disclose.

## AUTHORS’ CONTRIBUTIONS

C‐CH has received research support from Janssen, Merck, Gilead Sciences and ViiV and speaker honoraria from Abbvie, Bristol‐Myers Squibb, Gilead Sciences and ViiV, and served on advisory boards for Gilead Sciences, Janssen, ViiV and Abbvie. H‐YS has received research support from Gilead. Other authors have no competing interest to disclose.

C‐HK, H‐YS and C‐CH managed and supervised the study. S‐WL, C‐HK, H‐YS and C‐CH contributed to the study concept and design. C‐NC, Y‐TL, Y‐CC, K‐YL and W‐CL were involved in collection and assembly of clinical data. C‐NC and Y‐TL participated in the lab work. Y‐JL, K‐YL and H‐YS participated in the data analysis. B‐HL, C‐NC, C‐HK, H‐YS and C‐CH undertook interpretation of the data and drafted the report. All authors reviewed and approved the final version of the report.

## FUNDING

The study was supported by the grandts from Taiwan Centers for Disease Control (MOHW109‐CDC‐C‐114‐000108 and MOHW110‐CDC‐C‐114‐000103).

## Supporting information




**Table S1**. Fold changes of cytokine levels on Days 15 and 29 from their baseline for 48 people living with HIV and latent tuberculosis infection who received daily isoniazid plus rifapentine and coformulated bictegravir/emtricitabine/tenofovir alafenamide concurrently
**Table S2**. Clinical characteristics of the participants who failed to achieve plasma HIV RNA < 50 copies/ml on Days 15 and 29.
**Table S3**. Changes of isoniazid (INH) and rifapentine (RPT) concentrations after concurrent use with coformulated bictegravir/emtricitabine/tenofovir alafenamide
**Table S4**. Treatment completion rate and adverse events of 1‐month course of isoniazid plus rifapentine in 48 participants receiving coformulated bictegravir/emtricitabine/tenofovir alafenamide
**Table S5**. Solicited adverse events of 1‐month course of isoniazid plus rifapentine in 48 participants receiving coformulated bictegravir/emtricitabine/tenofovir alafenamide

## Data Availability

Deidentified participant‐level will be available on publication of the study. Requests for data should be sent to hysun@ntu.edu.tw and, on review of the proposed protocol and signing of a data sharing agreement, the data will be made available.
