# Supplementary material for: Short‐course daily isoniazid and rifapentine for latent tuberculosis infection in people living with HIV who received coformulated bictegravir/emtricitabine/tenofovir alafenamide
Source: J Int AIDS Soc. 2021 Nov 25;24(11):e25844. doi: 10.1002/jia2.25844 (PMC8614225; doi:10.1002/jia2.25844)
Supplement: Supplementary file 1 — Table S1. Fold changes of cytokine levels on Days 15 and 29 from their baseline for 48 people living with HIV and latent tuberculosis infection who received daily isoniazid plus rifapentine and coformulated bictegravir/emtricitabine/tenofovir alafenamide concurrently Table S2. Clinical characteristics of the participants who failed to achieve plasma HIV RNA < 50 copies/ml on Days 15 and 29. Table S3. Changes of isoniazid (INH) and rifapentine (RPT) concentrations after concurrent use with coformulated bictegravir/emtricitabine/tenofovir alafenamide Table S4. Treatment completion rate and adverse events of 1‐month course of isoniazid plus rifapentine in 48 participants receiving coformulated bictegravir/emtricitabine/tenofovir alafenamide Table S5. Solicited adverse events of 1‐month course of isoniazid plus rifapentine in 48 participants receiving coformulated bictegravir/emtricitabine/tenofovir alafenamide [file JIA2-24-e25844-s001.docx]

**Supplementary Table 1. Fold changes of cytokine levels on Days 15 and 29 from their baseline for 48 people living with HIV and latent tuberculosis infection who received daily isoniazid plus rifapentine and coformulated bictegravir/emtricitabine/tenofovir alafenamide concurrently**

| **Cytokines** | **Fold change on Day 15,**  **median (IQR; range)** | **Fold change on Day 29,**  **median (IQR; range)** |
| --- | --- | --- |
| **IFN-gamma** | 1.2 (1.0-1.6; 0.4-74.5) | 1.0 (0.8-1.2; 0.3-23.8) |
| **IL-10** | 1.3 (0.9-2.0; 0.04-25.0) | 1.1 (0.8-1.7; 0.04-116.1) |
| **IL-12p40** | 1.3 (0.8-1.9; 0.3-17.4) | 1.3 (1.0-2.0; 0.3-52.2) |
| **IL-12p70** | 1.1 (0.9-1.2; 0.4-5.1) | 1.0 (0.8-1.2; 0.3-2.0) |
| **IL-15** | 1.2 (0.9-1.5; 0.5-4.9) | 1.1 (0.9-1.3; 0.5-3.4) |
| **IL-17A** | 1.0 (0.8-1.2; 0.2-6.2) | 1.0 (0.8-1.2; 0.2-62.5) |
| **IL-2** | 1.1 (1.0-1.2; 0.7-7.0) | 1.0 (0.9-1.1; 0.6-135.7) |
| **IL-5** | 0.8 (0.5-1.4; 0.3-13.6) | 1.1 (0.9-1.2; 0.4-1.6) |
| **IL-6** | 1.1 (0.9-1.4; 0.3-73.7) | 1 (0.8-1.2; 0.3-3.5) |
| **IL-8** | 1.2 (0.9-1.6; 0.1-14.8) | 1.1 (0.8-1.6; 0.1-15.0) |
| **IP-10** | 1.3 (0.9-2.1; 0.3-21.3) | 1.1 (0.9-1.5; 0.3-5.3) |
| **MCP-1** | 1.2 (1.0-1.6; 0.4-3.4) | 1.2 (1.0-1.5; 0.6-3.1) |
| **MIP1B** | 1.3 (1.1-1.7; 0.3-4.0) | 1.3 (1.0-1.7; 0.3-3.0) |
| **TNF-alpha** | 1.2 (1.0-1.6; 0.3-5.3) | 1.2 (1.0-1.5; 0.3-3.2) |

**Abbreviations:** IFN, interferon; IL, interleukin; IP, interferon gamma-induced protein; IQR, interquartile range; MCP, monocyte chemoattractant protein; MIP, macrophage inflammatory protein; TNF, tumor necrosis factor

**Supplementary Table 2. Clinical characteristics of the participants who failed to achieve plasma HIV RNA <50 copies/ml on Days 15 and 29.**

| **No.** | **Age (years)/**  **Sex** | **Day 1**  **BW (kg)/BMI** | **Day 1**  **IQ** | **Day 1**  **PVL (copies/mL)** | **Day 15**  **IQ** | **Day 15**  **PVL**  **(copies/mL)** | **Day 29**  **IQ** | **Day 29**  **PVL**  **(copies/mL)** | **Month 3 PVL**  **(copies/mL)** | **Month 6 PVL**  **(copies/mL)** | **Month 12 PVL**  **(copies/mL)** |
| --- | --- | --- | --- | --- | --- | --- | --- | --- | --- | --- | --- |
| 1 | 40/M | 85/29 | 9.56 | <20 | 0.58 | 127 | 2.80 | <20 | <20 | 26 | 34 |
| 5 | 48/M | 81/28 | 47.47 | <20 | 2.30 | 64 | 0.70 | <20 | <20 | <20 | <20 |
| 7 | 44/M | 80/26 | 23.88 | <20 | 1.08 | 112 | 0.75 | <20 | <20 | <20 | NA |
| 10 | 38/M | 61/23 | 58.04 | <20 | 2.23 | <20 | 2.19 | 97 | <20 | <20 | NA |
| 13 | 72/M | 53/21 | 33.30 | 47 | 1.27 | 423 | 0.97 | <20 | 29 | <20 | 65.3 |
| 21 | 33/M | 72/23 | 12.56 | <20 | 0.78 | 216 | 0.87 | <20 | NA | <20 | NA |
| 22 | 46/M | 79/27 | 14.59 | <20 | 0.69 | <20 | 0.71 | 205 | <20 | <20 | <20 |
| 25 | 38/M | 55/19 | 29.37 | <20 | 1.50 | 315 | 0.95 | <20 | <20 | <20 | <20 |
| 28 | 58/M | 70/26 | 26.12 | <20 | 1.61 | 72 | NA | NA | <20 | <20 | NA |
| 29 | 38/M | 71/26 | 25.62 | <20 | 0.60 | 66 | 0.50 | <20 | <20 | <20 | NA |
| 34 | 38/M | 49/17 | 35.39 | <20 | 4.04 | 133 | 3.44 | <20 | <20 | <20 | <20 |
| 35 | 52/M | 64/24 | 14.71 | <20 | 6.58 | 127 | 6.29 | <20 | <20 | <20 | <20 |
| 36 | 54/M | 59/20 | 27.37 | <20 | 2.34 | 333 | 1.11 | <20 | <20 | <20 | <20 |
| 40 | 26/M | 57/21 | 12.91 | <20 | 0.89 | 52 | 0.99 | 32 | <20 | <20 | 71 |
| 41 | 53/M | 90/31 | 10.75 | <20 | 0.84 | <20 | 0.86 | 93 | <20 | <20 | NA |
| 42 | 38/M | 93/31 | 15.64 | <20 | 1.34 | 100 | 0.85 | 50 | <20 | <20 | NA |

**Note:** IQ, defined as the ratio of the BIC trough concentration to protein-adjusted 95% effective concentration (paEC_95_= 162 ng/mL)

**Abbreviations:** BIC, bictegravir; BMI, body-mass index; BW, body weight; IQ, inhibitory quotient; kg, kilogram; NA, not available; No., participant number; PVL, plasma HIV RNA load

**Supplementary Table 3. Changes of isoniazid (INH) and rifapentine (RPT) concentrations after concurrent use with coformulated bictegravir/emtricitabine/tenofovir alafenamide**

|  | Day 1 | Day 15 | Day 15 | Day 29 |
| --- | --- | --- | --- | --- |
| Drug | 3 hours after the 1^st^ dose | 24 hours after the 14^th^ dose | 3 hours after the 15^th^ dose | 24 hours after the 28^th^ dose |
| INH, median, IQR, µg/mL (n) | 0.72,  0.43-0.94  (44) | NA | 0.69  0.41-0.97  (42) | NA |
| RPT, median, IQR, µg/mL (n) | NA | 9.85,  6.82-13.43  (48) | NA | 9.88,  5.95-13.41  (46) |

**Abbreviations:** IQR, interquartile range; NA, not available

**Supplementary Table 4. Treatment completion rate and adverse events of 1-month course of isoniazid plus rifapentine in 48 participants receiving coformulated bictegravir/emtricitabine/tenofovir alafenamide**

| **Variable** | **1HP (n=48)** |
| --- | --- |
| Treatment completion rate, n (%) | 47 (97.9) |
| Discontinuation due to adverse events, n (%)^a^ | 1 (2.1) |
| Any adverse event, n (%)^b^ | 40 (83.3) |
| Drug-related adverse events, n (%) | 39 (81.3) |
| The maximal grade of adverse events, n (%)^c^ |  |
| Grade 1 | 18 (37.5) |
| Grade 2 | 14 (29.2) |
| Grade 3 | 8 (16.7) |
| Grade 4 | 0 (0) |
| Hospitalization, n (%)^d^ | 1 (2.1) |
| Death, n (%) | 0 (0) |

^a^One patient discontinued 1HP due to fever and generalized rashes on Day 15.

^b^The median duration of onset was 2 days (interquartile range, 1-11 days)

^c^Adverse events were graded according to common terminology criteria for adverse events (CTCAE).

^d^One patient was hospitalized due to influenza.

**Supplementary Table 5. Solicited adverse events of 1-month course of isoniazid plus rifapentine in 48 participants receiving coformulated bictegravir/emtricitabine/tenofovir alafenamide**

| **Adverse events** |  |
| --- | --- |
| Drug-related adverse events, n (%) | 39 (81.3) |
| Flu-like symptoms | 33 (68.8) |
| Fatigue | 26 (54.2) |
| Myalgia | 19 (39.6) |
| Headache | 18 (37.5) |
| Dizziness | 15 (31.3) |
| Arthralgia | 11 (22.9) |
| Fever | 6 (12.5) |
| Palpitation | 1 (2.1) |
| Cutaneous reaction | 22 (45.8) |
| Rash | 18 (37.5) |
| Itchiness | 4 (8.3) |
| Nausea | 11 (22.9) |
| Diarrhea | 7 (14.6) |
| Abdominal pain | 3 (6.3) |
| Insomnia | 3 (6.3) |
| Chest pain/tightness | 2 (4.2) |
| Rhinorrhea | 2 (4.2) |
| Sore throat | 2 (4.2) |
| Dry mouth | 2 (4.2) |
| Others* | 5 (10.4) |

*One participant each for productive cough, constipation, depressive mood, facial flushing, and tongue numbness.

**Supplementary section S-1. UHPLC-MS/MS method for analyzing bictegravir, isoniazid, and rifapentine concentrations**

The analytical procedure was performed using an Agilent 1290 UHPLC system coupled with an Agilent 6470 triple quadrupole system (Agilent Technologies, Waldbronn, Germany). An Agilent ZORBAX Eclipse Plus C18 2.1×100 mm (1.8 μm) column (Agilent Technologies, Waldbronn, Germany) was used for the separation. The mobile phase was a gradient of a mixture of 5 mM ammonium acetate in water (solvent A) and 0.1% formic acid in acetonitrile (solvent B). The gradient profile used was: 0-1 min, 10% solvent B (flow rate 0.3 mL min^-1^); 0.5 min, from 10% to 95% B (flow rate from 0.3 to 0.4 mL min^-1^); 3 min, 95% solvent B (flow rate 0.4 mL min^-1^); 1min, from 95% to 10% solvent B (flow rate from 0.4 to 0.3 mL min^-1^); 2.5 min 10% solvent B (flow rate 0.3 mL min^-1^). The sample reservoir and the column oven were maintained at 4^o^C and 25^o^C, respectively. The injection volume was 5 μL. Target drugs and their metabolites were analyzed in positive electrospray ionization mode. The ionization condition was performed as the following parameters: dry gas temperature of 350^o^C, dry gas flow rate of 11 L min^-1^, nebulizer pressure of 50 psi, sheath gas temperature of 350^o^C, sheath gas flow rate of 11 L min^-1^, nozzle voltage of 0 V, capillary voltage of 3500 V. The detection transition pair was set at m/z 138.1 → 121.0 for isoniazid, m/z 142.1 → 125.1 for isoniazid-d4, m/z 877.5 → 845.2 for rifapentine, m/z 885.5 → 151.1 for rifapentine-d8, m/z 450.1 → 289.1 for bictegravir, and m/z 453.1 → 289.1 for bictegravir-^15^N, d2, respectively.

**Supplementary section S-2. UHPLC-MS/MS method for analyzing tenofovir and emtricitabine concentrations**

The analytical procedure was performed using an Agilent 1290 UHPLC system coupled with an Agilent 6460 triple quadrupole system (Agilent Technologies, Waldbronn, Germany). An ACQUITY HSS T3 2.1×100 mm (1.8 μm) column (Waters, Milford, MA, U.S.) was used for the separation. The mobile phase was a gradient of a mixture of 0.1% formic acid in water (solvent A) and 0.1% formic acid in acetonitrile (solvent B). The gradient profile used was: 0-2 min, 0.5% solvent B; 2-4 min, from 0.5% to 50% B; 4-5.5 min, from 50% to 95% solvent B; 5.5 to 6.5 min, from 95% to 0.5% solvent B; 6.5 to 7.5 min 0.5% solvent B. The flow rate was set at 0.25 min mL^-1^. The injection volume was 5 μL. Target drugs were analyzed in positive electrospray ionization mode. The ionization condition was performed as the following parameters: dry gas temperature of 300^o^C, dry gas flow rate of 6 L min^-1^, nebulizer pressure of 45 psi, sheath gas temperature of 325^o^C, sheath gas flow rate of 11 L min^-1^, nozzle voltage of 500 V, capillary voltage of 3500 V. The detection transition pair was set at m/z 288 → 175 for tenofovir, m/z 294 → 182 for tenofovir-d5, m/z 248 → 129 for emtricitabine, and m/z 251 → 133 for emtricitabine-^13^C^15^N_2_, respectively.
